# Transcriptome analysis under pecan scab infection reveals the molecular mechanisms of the defense response in pecans

**DOI:** 10.1371/journal.pone.0313878

**Published:** 2024-11-21

**Authors:** Gaurab Bhattarai, Hormat Shadgou Rhein, Avinash Sreedasyam, John T. Lovell, Sameer Khanal, Jane Grimwood, Jeremy Schmutz, Jerry Jenkins, Peng W. Chee, Cristina Pisani, Jennifer Randall, Patrick J. Conner

**Affiliations:** 1 Institute of Plant Breeding, Genetics & Genomics, University of Georgia, Athens, Georgia, United States of America; 2 Department of Entomology, Plant Pathology and Weed Science, New Mexico State University, Las Cruces, New Mexico, United States of America; 3 Genome Sequencing Center, HudsonAlpha Institute for Biotechnology, Huntsville, Alabama, United States of America; 4 US Department of Energy Joint Genome Institute, Berkeley, California, United States of America; 5 Department of Crop and Soil Sciences, University of Georgia-Tifton Campus, Tifton, Georgia, United States of America; 6 U.S. Department of Agriculture (USDA), Agricultural Research Service (ARS), Southeastern Fruit and Tree Nut Research Station, Byron, Georgia, United States of America; 7 Department of Horticulture, University of Georgia-Tifton Campus, Tifton, Georgia, United States of America; Jeju National University, REPUBLIC OF KOREA

## Abstract

Pecan scab, caused by the fungal pathogen *Venturia effusa*, is the most devastating disease of pecan (*Carya illinoinensis*) in the southeastern United States. Resistance to this pathogen is determined by a complex interaction between host genetics and disease pathotype with even field-susceptible cultivars being resistant to most scab isolates. To understand the underlying molecular mechanisms of scab resistance in pecan, we performed a transcriptome analysis of the pecan cultivar, ‘Desirable’, in response to inoculation with a pathogenic and a non-pathogenic scab isolate at three different time points (24, 48, and 96 hrs. post-inoculation). Differential gene expression and gene ontology enrichment analyses showed contrasting gene expression patterns and pathway enrichment in response to the contrasting isolates with varying pathogenicity. The weighted gene co-expression network analysis of differentially expressed genes detected 11 gene modules. Among them, two modules had significant enrichment of genes involved with defense responses. These genes were particularly upregulated in the resistant reaction at the early stage of fungal infection (24 h) compared to the susceptible reaction. Hub genes in these modules were predominantly related to receptor-like protein kinase activity, signal reception, signal transduction, biosynthesis and transport of plant secondary metabolites, and oxidoreductase activity. Results of this study suggest that the early response of pathogen-related signal transduction and development of cellular barriers against the invading fungus are likely defense mechanisms employed by pecan cultivars against non-virulent scab isolates. The transcriptomic data generated here provide the foundation for identifying candidate resistance genes in pecan against *V*. *effusa* and for exploring the molecular mechanisms of disease resistance.

## Introduction

Pecan scab, caused by the plant pathogenic fungus *Venturia effusa* (G. Winter) Rossman & W.C. Allen (syn. *Fusicladium effusum*), is the most damaging disease of pecan in the southeastern United States. Under favorable environmental conditions and in the presence of a susceptible host, pecan scab can cause severe yield loss and reduction in nut quality [1]. For example, in the 2018 season, under moderate to high scab pressure, the reduction in crop yield in the state of Georgia was estimated to be 12% [2]. Current scab management practices depend heavily on the intensive use of conventional fungicides, which has led to the development of fungicide resistance in *V*. *effusa* [3, 4]. On average, 10 to 14 fungicide sprays are needed per year to adequately control pecan scab in susceptible cultivars [2]. Fungicides are applied beginning in early spring as the developing leaves are susceptible to the primary infection from the overwintered lesions on the previous season’s shoots and shucks. While mature leaves are resistant to scab infection, frequent sprays (every two weeks or more frequently) may be needed until shell hardening if environmental conditions favor disease development (frequent rains), as nut shucks remain susceptible throughout the growing season. Intensive use of fungicides not only increases the cost of production but also seriously threatens the ecosystem and can affect the health of humans and animals. The most sustainable approach to managing pecan scab is to utilize the host’s genetic resistance to infection. The development of scab-resistant cultivars has been a primary goal of the pecan breeding programs at the University of Georgia and the United States Department of Agriculture [5].

The presence of pathogenic variability adds complexity to resistance breeding efforts. In a study where 19 pecan cultivars were inoculated with 12 monoconidial scab isolates collected from 8 cultivars at 7 different locations, it was found that scab isolates were most virulent on cultivars from their respective origins and a few others. These results suggest that a large pool of differential resistance exists in pecan germplasm and that scab resistance is as dependent upon the pathotype of the challenging scab isolate as the host genetic makeup [6]. Some cultivars exhibit resistance to the most common isolates, providing satisfactory field resistance, sometimes for decades, but this resistance can be lost with the appearance of new virulent pathotypes. Thus, most pecan cultivars are capable of displaying both resistant and susceptible interactions with the scab pathogen, and levels of field susceptibility are governed by the availability of virulent pathotypes within the orchard. Molecular marker analysis revealed high genetic diversity among scab pathogen populations from different geographical regions in the southern United States [7]. Bock et al. (2022) provided some molecular evidence of a population structure within scab pathogens between orchards of a single cultivar based on the physical distance between them, but there was limited evidence of such structure between pathogen populations on different trees of the same cultivar within an orchard [8]. Recent findings, including the demonstration of the sexual stage *in vitro* [9] and the observation of equilibrium in mating types [10], strongly support the presence of a sexual cycle in *V*. *effusa*, though it remains unidentified in the field [7]. These observations underscore the need for a comprehensive understanding of pathotype-specific scab resistance mechanisms to facilitate the development of pecan cultivars with durable resistance to the scab pathogen.

The understanding of scab resistance in pecans at the molecular level is limited. Very few genetic studies have been carried out to map scab resistance and identify candidate genes. Quantitative trait locus (QTL) mapping in a biparental mapping population has shown a single QTL region at linkage group 12 with two overlapping QTLs for scab incidence in ‘Elliott’ [11]. Identification of this QTL region was based on the assessment of native disease on pecan leaves in the field in consecutive years and may be caused by the same or different pathogen variants. A third year of observations yielded no significant QTLs. These differences highlight the difficulty of performing field studies of disease resistance where environmental and pathotype diversity cannot be controlled. Xiao et al. (2021) tested the expression of genes involved in chitin signaling pathways in chitin-treated leaves of the pecan cultivars Excel and Pawnee and reported a possible involvement of these genes in recognizing chitin residue of fungal hyphae fragmented by the host’s chitinase enzyme [12]. In the closely related pathogen *Venturia inaequalis* (cause of apple scab), molecular characterization of host resistance has shown receptor kinase coding genes to be involved in signal reception by the extracellular domain of transmembrane proteins and transduction of these signals into the cell via phosphorylation activities by the intracellular kinase domain [13, 14]. Successful intergenic transfer of the apple scab resistance receptor-like protein (RLP) gene (*Rvi6*) into pear has shown evidence of resistance against European pear scab (caused by *V*. *pyrina*) [15]. However, resistance from *Rvi6* in pear showed variability according to the strain of *V*. *pyrina* used as one of the strains tested overcame the resistance of most of the transgenic pear clones. This clearly signals that the scab resistance in apples is pathogen-specific and genetically diverse host genotypes can have allelic variation in resistance genes to confer resistance against diverse pathogenic isolates.

In this research, we studied the transcriptional response of the pecan cultivar ‘Desirable’ when challenged with two scab isolates with different pathogenicity. ‘Desirable’ was commercially introduced in 1945, and it is one of the most widely planted pecan cultivars in the southeastern U.S. [16]. ‘Desirable’ produces large, well-filled nuts with excellent kernels and a high complete-halves shelling percentage. In the first decades after release, ‘Desirable’ was resistant to the most common pathotypes and was considered resistant to pecan scab [16]. In recent years, however, the buildup of virulent pathotypes in commercial orchards has resulted in ‘Desirable’ becoming one of the most scab-susceptible pecan cultivars, requiring intensive fungicide protection throughout the season [17]. This situation mirrors the loss of resistance seen in most other pecan cultivars that have been widely planted by the industry in scab favorable environments [6, 16]. To capture the real-time transcriptional response, we performed RNA sequencing (RNA-Seq) and differential gene expression analysis of inoculated leaf samples at three different time points: 24, 48, and 96 hours post-inoculation (hpi). We also performed weighted gene co-expression network analysis to highlight correlated gene networks related to defense response. The study reveals temporal differences in the gene expression pattern and molecular mechanisms involved in the resistance and susceptibility of pecans to the scab fungus. The results of this study lay the foundation for understanding the molecular mechanism and pathways, as well as the identification of candidate genes involved in conferring resistance to pecan scab in the future.

## Materials and methods

### Plant material and fungal isolates

The pecan cultivar ‘Desirable’ was used for inoculation with *V*. *effusa* and subsequent RNA-Seq analysis. Pecan trees are propagated by grafting scion wood of the chosen cultivar onto open pollinated seedling rootstocks. Dormant, bare-root ‘Desirable’ trees were obtained from a nursery and planted into a 6.23-liter tree pot (TP616, Stuewe & Sons, Tangent OR USA) in a pine bark and peat growing medium. Two *V*. *effusa* isolates were chosen for use in the experiment. The first isolate, De-Tif-11, was obtained from a single conidium extracted from a lesion produced on a ‘Desirable’ nut collected from a Tift Co., GA orchard (USA). Previous experiments indicated that this isolate sporulated well in culture and readily infected ‘Desirable’ leaves. The second isolate, Pa-OK-11, was cultured from a single conidium obtained from a ‘Pawnee’ nut collected from an orchard in Payne, OK, USA. ‘Desirable’ was previously shown to be highly resistant to Pa-OK-11 [6]. After collection, *V*. *effusa* were stored, and cultured for conidial production as previously described [18]. In brief, frozen stocks of germinated conidia (stored at -20°C) were initially revived by placing filter paper discs with dried germinated conidia directly onto 5% oatmeal agar plates containing the antibiotics streptomycin, chloramphenicol, and tetracycline at a concentration of 50 g·L^-1^. These plates were then incubated in a growth chamber at 24°C under a 12 h light period provided by fluorescent lights for two weeks. Fungal colonies were subsequently homogenized in sterile water and spread onto fresh oatmeal agar plates, which were again incubated for two weeks. Subsections of these plates were homogenized in sterile water and spread onto oatmeal agar plates to produce conidia for inoculations. After 1 to 2 weeks of growth, conidia were harvested, and their concentration was adjusted to 1 × 10^6^ conidia/mL of distilled water for inoculation purposes.

### Inoculation and disease assessment

Trees were inoculated in May 2019 on the first flush of leaf growth from the trees. Trees were prepared for inoculation by selecting the most susceptible leaflets, those ranging from 1/3 to 2/3 full expansion [18]. All other leaflets were removed. Since a single tree did not produce a sufficient number of leaves to sample all time points, each biological replication consisted of a group of five trees. Each of the three treatments, isolate De-Tif-11, isolate Pa-OK-11, and water control, was applied to three replications of five trees. Thus, each treatment was applied to 15 trees, and a total of 45 trees were used in the experiment. Leaves of trees in the control group (group C) were mock-inoculated with sterilized diH_2_O, and the other two treatment groups were sprayed until run off with a conidial suspension of scab isolates Pa-OK-11 (group R) and De-Tif-11 (group S) (1 × 10^6^ conidia/mL). After inoculation, trees were placed in a humidity room at 24–27°C (sealed room with overhead light and several humidifiers running) to maintain free moisture on leaf surfaces for 48 h. Subsequently, trees were removed and placed in a warehouse with diffuse overhead light provided by interspersed clear ceiling panels (12 h day length, ambient humidity, 20–29°C) for the remainder of the experiment. Two leaflets from each tree were collected and frozen with liquid nitrogen at 24, 48, and 96 hours post-inoculation (hpi). Thus, for each treatment, there were 3 biological replicates (n = 3), each containing 10 leaflets (2 each from 5 trees). In addition, four leaflets were collected from each biological replicate at 72 hpi and stained following the previously published staining method [18] to visualize and quantify the fungal infection and the resistance reaction in inoculated leaf tissue.

### RNA isolation, sequencing, reads alignment and differential gene expression analysis

Total RNA was isolated and purified from the leaf tissues using a Norgen Plant/Fungi Total RNA Purification Kit (Norgen Biotek Corp., Tharold, Ontario, Canada). Paired-end sequencing (150-bp read length) was performed using Illumina HiSeq platform (Illumina, San Diego, CA). Raw reads were checked for quality with FastQC v0.11.8 (http://www.bioinformatics.babraham.ac.uk/projects/fastqc/), and adapters trimmed, and filtered for minimum read quality (30) and minimum length (36 bp) using Trim Galore v0.6.5 (https://www.bioinformatics.babraham.ac.uk/projects/trim_galore/). Processed reads were first aligned against the SILVA rRNA database for eukaryotes [19] using Bowtie2 v2.4.1 [20] to remove any ribosomal RNA reads present. Unaligned paired reads were recovered and aligned against the ‘Pawnee’ reference genome [21] using STAR v2.7.3 [22] with default parameters (S1 File). Read counts per gene were obtained using HTseq v0.13.5 [23]. Linear differential gene-expression analysis was performed via Wald contrasts with DESeq2 v1.28.190 [24]. Differentially expressed genes were defined as those with Benjamini-Hochberg adjusted contrast *P*-value ≤ 0.05 and |log2 fold-change| ≥ 1.5. To construct the heatmaps of selected differentially expressed genes, Euclidean distance between genes were calculated, and genes were clustered using the ‘complete’ method. Heatmaps were generated using ‘pheatmap’ [25] package in R.

### Gene ontology enrichment and weighted gene co-expression network analysis

Differentially expressed genes at each time point were subjected to gene ontology enrichment analysis using Fisher’s exact test in topGO v2.40.091 [26]. GO terms with Fisher’s exact test of *P*<0.05 were considered significant. To construct the network of genes with correlated expression pattern, a weighted gene co-expression network analysis (WGCNA) was performed using the R package WGCNA [27]. The normalized read counts for all differentially expressed genes at all three time points in the resistant and susceptible reactions was obtained from DESeq2 normalization step and combined. Then, the dataset was refined to eliminate duplicate genes, creating a unique set of gene IDs differentially expressed in at least one of the sample comparisons. A one-step function of network construction was performed, and consensus modules (a group of DE genes with a highly similar expression pattern) were detected. An unsigned topological overlap matrix (TOM) was generated to calculate a soft threshold power. The network was subsequently constructed using a soft threshold power of 16, module size 30 (minimum number of genes to have in a cluster), and tree cut height of 0.25. Gene ontology enrichment analysis was performed for genes present in each module to investigate the functional roles of these co-expressed genes.

### Gene network construction and hub gene selection

Weighted gene co-expression networks obtained from WGCNA are composed of nodes (genes) and edges (co-expression relationship with other genes which is determined by the pairwise correlations between gene expressions). Among several algorithms available to estimate the interaction level of each node to another, the ‘Degree’ method is based on the ranking of nodes based on the number of edges connected to them. This method proposes that the more nodes connected to a certain node the more likely it plays an important role in a gene network [28]. We identified the top 50 genes (hub genes) with the highest interaction from two gene modules (M3 and M4) significantly enriched for GO terms related to defense response and response to biotic stimulus. The gene network was uploaded to Cytoscape v3.9.0 [29], and a plugin cytoHubba [30] was used to identify the top 50 genes using the ‘degree’ method.

### Quantitative real-time PCR (RT-qPCR)

Forward and reverse primers for 11 differentially expressed defense-related genes were designed using the coding sequence of corresponding genes with Geneious Prime v2022.0.1 (https://www.geneious.com) (S1 Table). Total RNA was isolated and purified from the same leaf tissues used for RNA sequencing using the same extraction and purification protocol described above. Quantity and purity of extracted RNA were measured using Take3^™^ Multi-Volume Plate (BioTek Inc., Winooski, VT, USA). Single-stranded complementary DNA (cDNA) was synthesized using a reverse transcription (RT) reaction following the manufacturer’s instructions (High-capacity RNA-to-cDNA Kit, Applied Biosystems, catalog # 4387406). RNA (1 μg) was taken and mixed with 10 μL 2X RT buffer mix, 1 μL 20X RT enzyme mix, and the remaining volume of nuclease-free H_2_O (Q.S., quantity sufficient) to make a final reaction volume of 20 μL. The RT reaction mix was prepared on ice. Thermal cycling conditions for the reverse transcription reaction were: 37°C for 60 min. followed by 95°C for 5 min., and a final hold at 4°C. Synthesized cDNA was diluted to a final volume of 100 μL (1 ng/μL) with nuclease-free H_2_O and stored at -20°C until use for RT-qPCR. RT-qPCR was performed in a StepOne^™^ Real-Time PCR System (Applied Biosystems). The 10 μL reaction mixture consisted of 1 ng of single-stranded cDNA (2 μL of 0.5 ng/μL), 0.5 μL of each forward and reverse primers (0.1 μM), 5 μL iTaq Universal SYBR Green Supermix (Bio-Rad Laboratories, USA), and 2 μL of nuclease-free water. The reaction was run with the following thermal cycles: 95°C for 10 min., followed by 40 cycles of 95°C for 15 sec. and 54°C for 30 sec. Melting curve data was generated by adjusting a temperature gradient ranging from 60°C to 95°C with plate reads at every temperature increment of 0.3°C. The housekeeping gene *ACTIN* was used to normalize the expression of candidate genes using the 2^ΔΔCt^ method [31].

## Results

### Quantification of scab resistance

**Fig 1:**
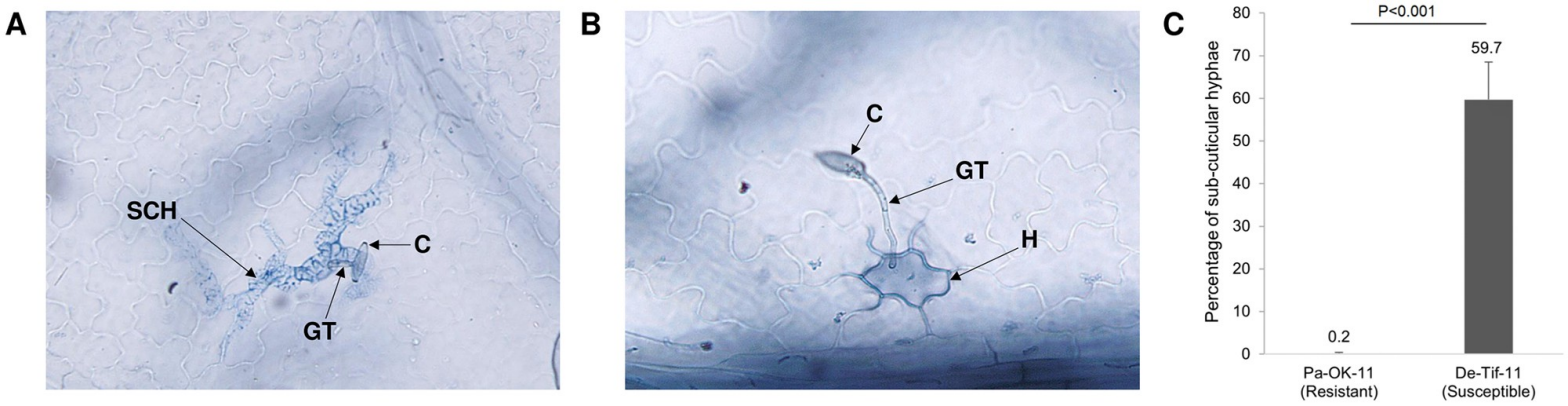
Visualization of *Venturia effusa* infection under microscope. (A) Susceptible and (B) resistant reactions in ‘Desirable’ leaf tissue to scab fungus (*V*. *effusa*) infection viewed under a light microscope (400x magnification). C: conidia, GT: germ tube, H: halo (indication of resistant reaction), SCH: sub-cuticular hyphae (indication of a susceptible reaction). (C) Average percentage of subcuticular hyphae produced by conidia counted in ‘Desirable’ inoculated with pathogenic (De-Tif-11), and non-pathogenic (Pa-OK-11) scab isolates. *P*<0.001 indicates a significant difference between two isolates for average percentage of sub-cuticular hyphae.

The susceptibility of the ‘Desirable’ leaf samples inoculated with the two scab isolates, De-Tif-11 (pathogenic) and Pa-OK-11 (non-pathogenic), was quantified by calculating the percentage of germinated conidia producing sub-cuticular hyphae (SCH) [32]. Successful fungal infections involved conidial germination (producing a germ tube), penetration into the subcuticular space using an appressorium and subsequent development of subcuticular hyphae (Fig 1). Resistance reactions were identified as halo structures around penetration sites which indicates the stoppage of fungal spread by the host (Fig 1). At 72 hpi, the De-Tif-11 isolate had produced SCH at 59.7% of the germinated conidia while the Pa-OK-11 isolate had produced SCH on only 0.2% of the germinated conidia. The mean percentage of SCH was significantly higher (*P*<0.001) in the leaf samples receiving De-Tif-11 inoculation (susceptible reaction) compared to Pa-OK-11 inoculation (resistant reaction) (Fig 1). No fungal conidia were observed in control samples which were mock inoculated with diH_2_O.

### RNA sequencing, alignment statistics, and profile of gene expression

**Fig 2:**
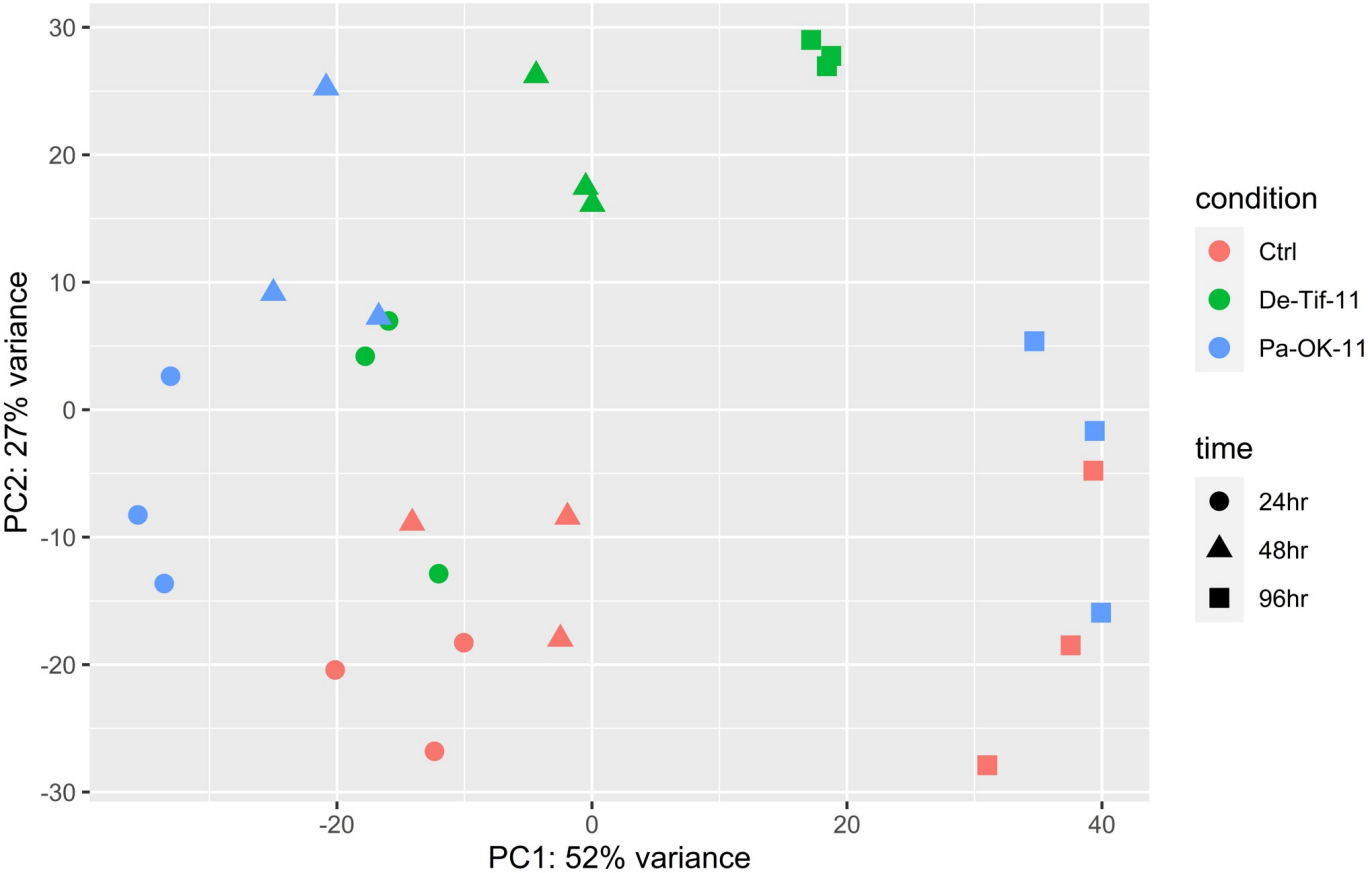
Principal component analysis (PCA) of variance stabilization transformed (vst) normalized gene expression values in ‘Desirable’. Each dot represents a biological replicate of the treatment (leaf samples inoculated with a non-pathogenic Pa-OK-11 or a pathogenic De-Tif-11 scab isolate) and control (H_2_O inoculated) sample. First (PC1) and second (PC2) principal components explained 52 and 27% of total variance in the gene expression across samples, respectively. Ctrl = control, De-Tif-11 = susceptible reaction, Pa-OK-11 = resistant reaction, hpi = hours post inoculation, 24hr = 2h hpi, 48hr = 48 hpi, 98hr = 96 hpi.

A total of 2.06 billion raw paired-end short reads were obtained from the sequencing of the 27 samples (S2 Table). Raw reads varied from 21,476,723 to 156,952,681 read pairs per sample (average of 76,345,360 read pairs). On average, 2.68% of raw read pairs were discarded in quality filtering. Additionally, 13.2% of quality reads were aligned to the SILVA ribosomal RNA database and reads derived from rRNA were thus removed from downstream analysis. This left an average of 65,719,020 read pairs per sample to map against the pecan reference genome (REF). Of these reads, 85.2–94.3% mapped uniquely to the reference genome (average 91.4%), indicating good quality of the sequence data. Principal component analysis (PCA) using all three biological replicates of each sample shows a large percentage of variation between samples was contributed by the time factor, and a smaller but also significant proportion of variation was due to the scab isolate (Fig 2). This indicates gene expression differences after inoculation are affected by both the time after inoculation and the genotype of the fungal isolate. There was a clear separation in gene expression among treatments at 48 hpi suggesting that early molecular responses might be important in conferring resistance to *V*. *effusa*. However, by 96 hpi, the resistant and control treatments clustered close to each other and away from the susceptible treatment. Thissuggests the earlier molecular defense responses in the resistant reaction were sufficient to control pathogen growth and the host is return to normal expression patterns. Contrastingly, in the susceptible reaction, further growth of the pathogen results in continued differential gene expression.

#### Pattern of differential gene expression in resistance and susceptible reaction

**Fig 3:**
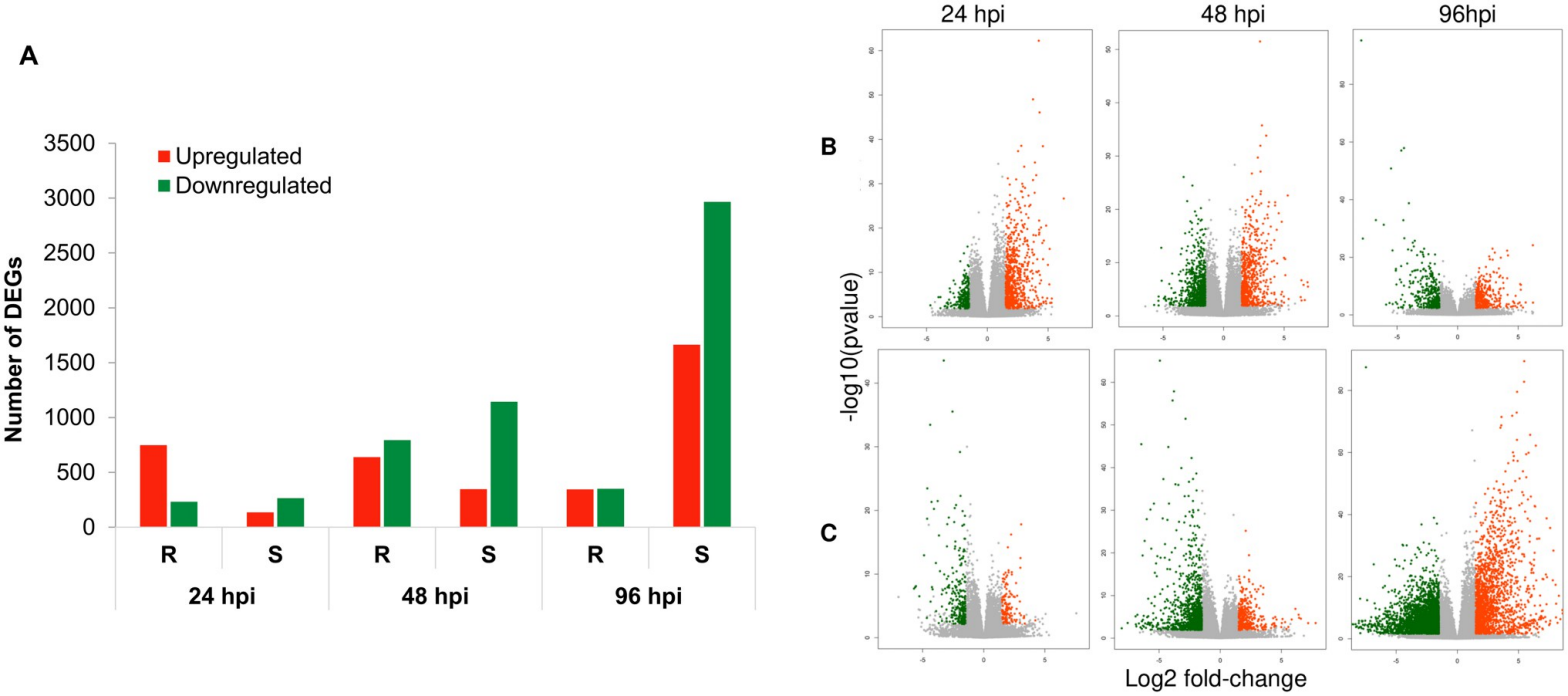
Differentially expressed genes in pecan cultivar Desirable. A) Bar graph showing the number of up and downregulated genes in susceptible (inoculated with a pathogenic monoconidial *V*. *effusa* isolate, De-Tif-11) and resistant (inoculated with a non-pathogenic monoconidial *V*. *effusa* isolate, Pa-OK-11) reactions in ‘Desirable’ at 24, 48, and 96 hours post inoculation (hpi). B, C) Volcano plot showing differentially expressed genes (DEGs) in resistant (B) and susceptible (C) reaction. Red and green dots represent upregulated and downregulated genes (|log2 fold-change| ≥ 1.5, false discovery rate (FDR)-adjusted *P*-value threshold<0.05).

**Fig 4:**
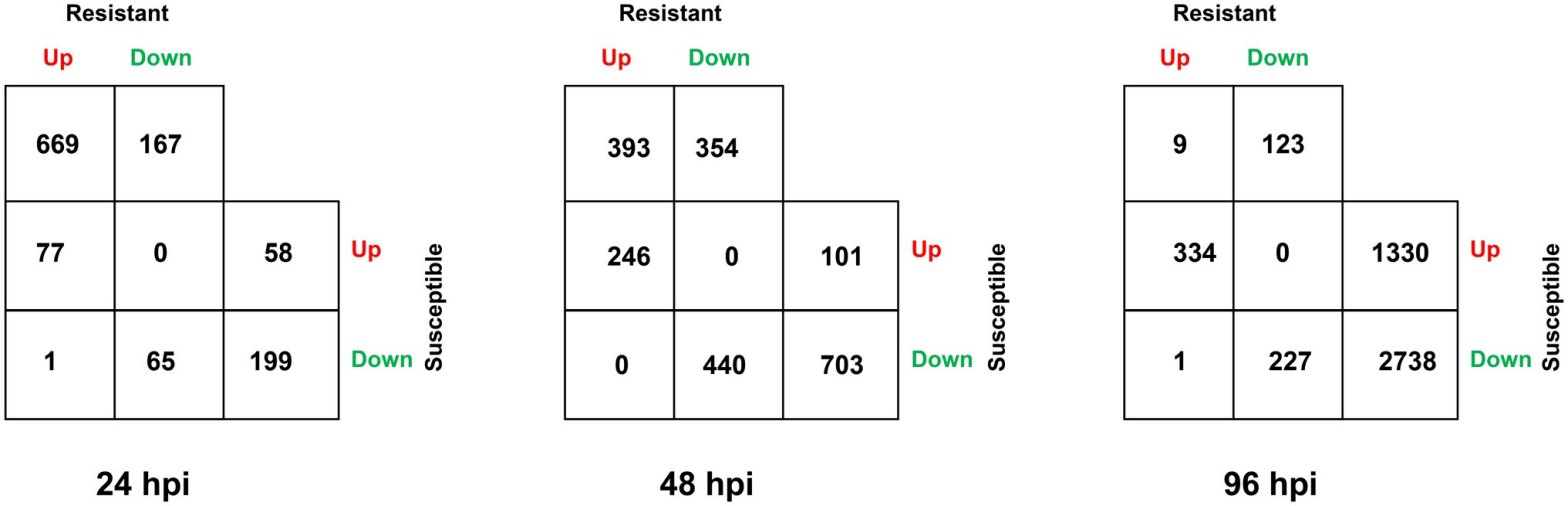
Number of up and downregulated genes shared between resistant and susceptible reactions in ‘Desirable’ at 24, 48 and 96 hours post inoculation (hpi). Resistant and susceptible reactions refer to the treatment group challenged with a non-pathogenic (Pa-OK-11) or a pathogenic (De-Tif-11) monoconidial *V*. *effusa* isolate, respectively.

Differentially expressed genes at each time point were identified by statistically comparing the mean level of gene expression in susceptible or resistant reaction to that in control (mock inoculated samples). The number of differentially expressed genes (DEGs) (Benjamini-Hochberg adjusted contrast *P*-value ≤ 0.05 and |log2 fold-change| ≥ 1.5) varied markedly between the resistant and susceptible reactions at all three different time points (Fig 3, Table 1, S3–S8 Tables). The number of upregulated genes (log2 fold-change ≥ 1.5) was highest in the resistant reaction at 24 hpi (747) and decreased progressively at 48 hpi (639) and 96 hpi (344) (Table 1). In contrast, the opposite pattern was detected in the susceptible reaction where only 135 genes were upregulated at 24 hpi with a rapid increase at 48 (347) and 96 hpi (1664). A similar pattern of rapid increase occurred in the susceptible reaction for the number of downregulated genes. The number of downregulated (log2 fold-change ≤ -1.5) genes increased from 265 at 24 hpi to 1,143 and 2,966 at 48 and 96 hpi, respectively. In the resistant reaction, however, the peak for the number of downregulated genes was obtained at 48 hpi (794), which was more than twice the number of downregulated genes found at 24 (232) and 96 hpi (350). There was a single DEG that was upregulated in resistant reactions and downregulated in susceptible reactions at 24 (CiPaw.12G074800) and 96 hpi (CiPaw.03G214500) (Fig 4), and no genes were downregulated in resistant and upregulated in susceptible reactions at any time points. Therefore, almost all DEGs that were common between resistant and susceptible reactions at any time points were expressed differentially in the same direction. However, the number of these common genes significantly varied at the three different time points. Compared to 24 hpi (10.3%), proportionately more genes that were upregulated in the resistant reaction were common with genes upregulated in the susceptible reaction at later time points (38.3% at 48 hpi and 97.1% at 96 hpi).

**Table 1:** Number of up-and down-regulated genes at three different time points in pecan leaflets resistant or susceptible to infection by a pathogenic or a non-pathogenic isolate of *Venturia effusa*.

Time points	Resistant reaction (Pa-OK-11 vs Control)			Susceptible reaction (De-Tif-11 vs Control)		
	*Upregulated*	*Downregulated*	*Total*	*Upregulated*	*Downregulated*	*Total*
24 hpi	747	232	979	135	265	400
48 hpi	639	794	1433	347	1143	1490
96 hpi	344	350	694	1664	2966	4630

### Gene ontology enrichment revealed pathways related to defense mechanisms

**Fig 5:**
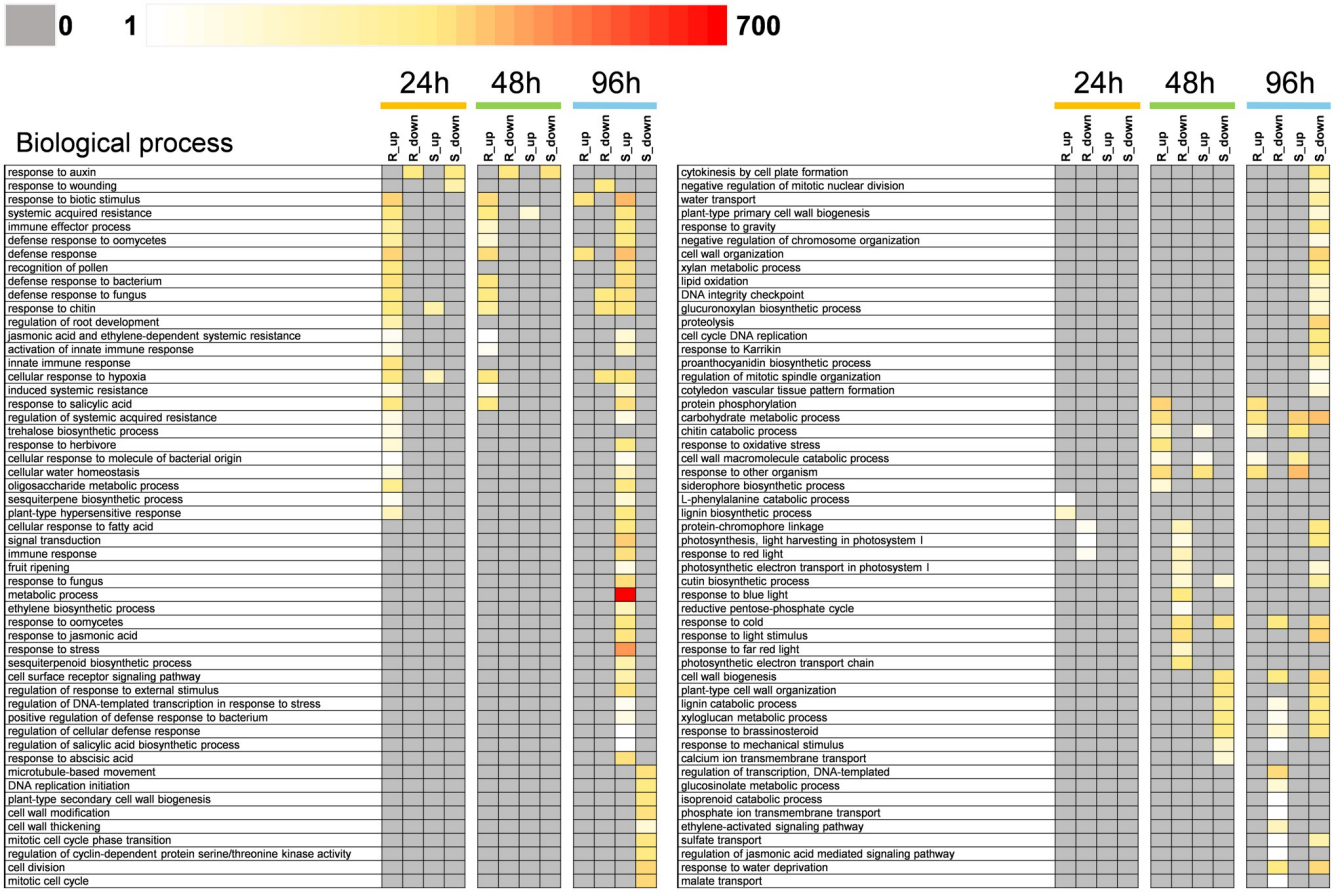
Significantly enriched biological processes (BP) observed in resistant and susceptible reaction in ‘Desirable’ at three different time points (24, 48, 96 hours post inoculation (hpi)). Resistant and susceptible reactions refer to the treatment group challenged with a non-pathogenic (Pa-OK-11) or a pathogenic (De-Tif-11) monoconidial *V*. *effusa* isolate, respectively. Color intensity represents the number of up (log2fold-change ≥ 1.5) or downregulated genes (log2fold-change ≤ -1.5) related to corresponding biological processes. R_up and R_down: up and downregulated genes in resistant reaction, S_up and S_down: up and downregulated genes in susceptible reaction, respectively.

Gene ontology (GO) enrichment analysis was performed for both upregulated and downregulated genes at each time point in susceptible and resistant reactions. Gene ontology enrichment analysis showed upregulated genes in the resistant reaction at 24 and 48 hpi are primarily involved in biological processes related to defense responses. At both time points, biological processes such as response to biotic stimulus, system acquired resistance, immune effector process, defense response to oomycetes, defense response, defense response to fungus, and response to chitin were significantly enriched and associated with upregulated genes (Fig 5). Among them, biological processes: response to biotic stimulus and defense response were consistently enriched at all three time points. Most of these biological processes were not represented by either up-and downregulated genes in the susceptible reaction at 24 and 48 hpi. However, these biological processes related to defense response only appeared later (96 hpi) in the susceptible reaction where they attributed to the upregulated genes. These differential GO patterns between the susceptible and resistant reactions may indicate the expression of defense-related genes at an early stage of fungal infection is a key molecular mechanism of scab resistance, whereas expression of these same genes at a later time may be insufficient to enable resistance. Among the biological processes that were only upregulated in the 24 hpi resistant reaction were the L-phenylalanine catabolic process and lignin biosynthetic process. The L-phenylalanine catabolic process is regulated by phenylalanine lyase genes and plays an important role in biosynthesis of lignin particularly by deamination of phenylalanine to form trans-cinnamic acid [33, 34]. Upregulation of genes related to biosynthesis of cellular components like lignin can be key in providing a physical barrier against the developing fungal structures on the cell surface. A contrasting pattern of GO enrichment was observed in the susceptible reaction at 96 hpi. In susceptible reaction at 96 hpi, genes related to biological processes such as cell wall thickening, cell wall modification, plant-type primary and secondary cell-wall biogenesis, and cell wall organization were downregulated. However, none of these GO terms were significantly enriched at any other time points in either resistant or susceptible reactions. The downregulation of genes related to primary and secondary cell wall biogenesis and cell wall thickening may indicate the compromised state of cell wall and cell wall components of the host cells and the importance of maintaining rigid cell wall structure to confer resistance against the growing fungus.

### Weighted gene-co expression network analysis

**Fig 6:**
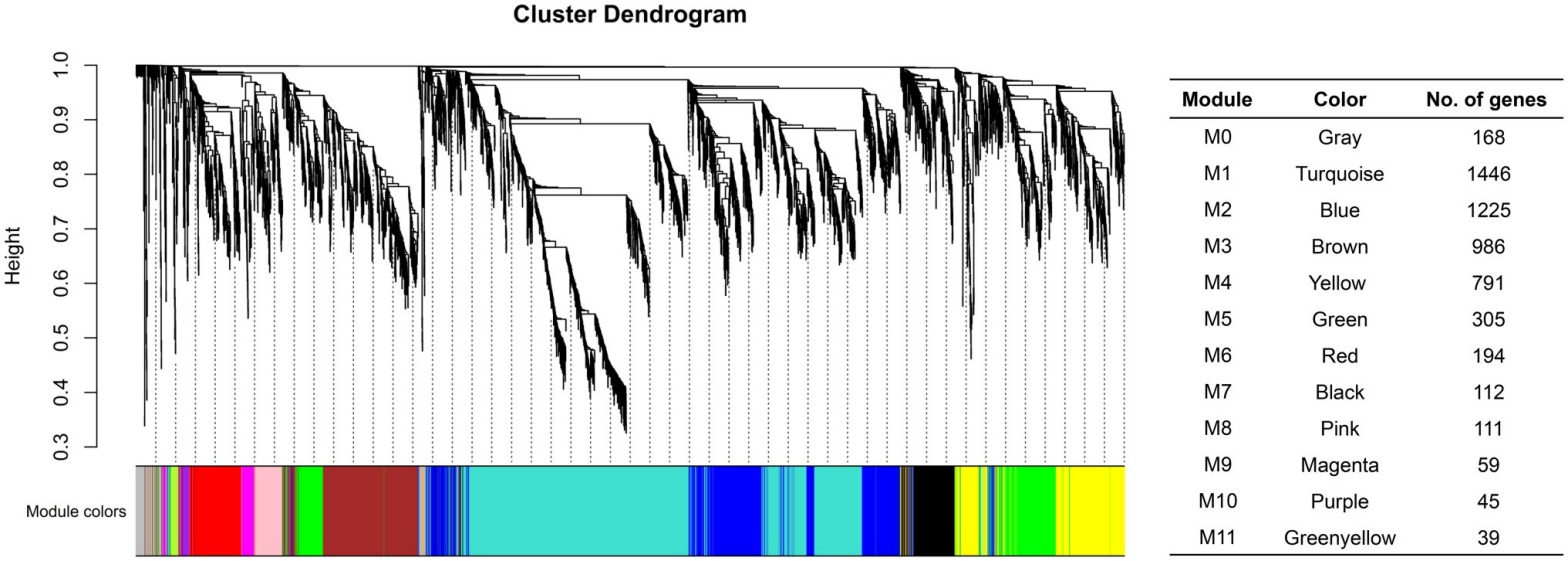
WGCNA hierarchical clustering tree. Dendrogram showing 11 different gene clusters (modules) consisting of differentially expressed genes with similar expression pattern in Desirable cultivar of pecan when challenged with a pathogenic (De-Tif-11) or a non-pathogenic (Pa-OK-11) monoconidial *V*. *effusa* isolate. The gene expression pattern is bidirectional (each gene pair can have both upregulated, both down regulated or one upregulated and other downregulated pattern of gene expression) and table on the right shows number of genes in each module. Each color in cluster dendrogram represents a module. Gray module (M0) has genes not assigned to any gene modules.

**Fig 7:**
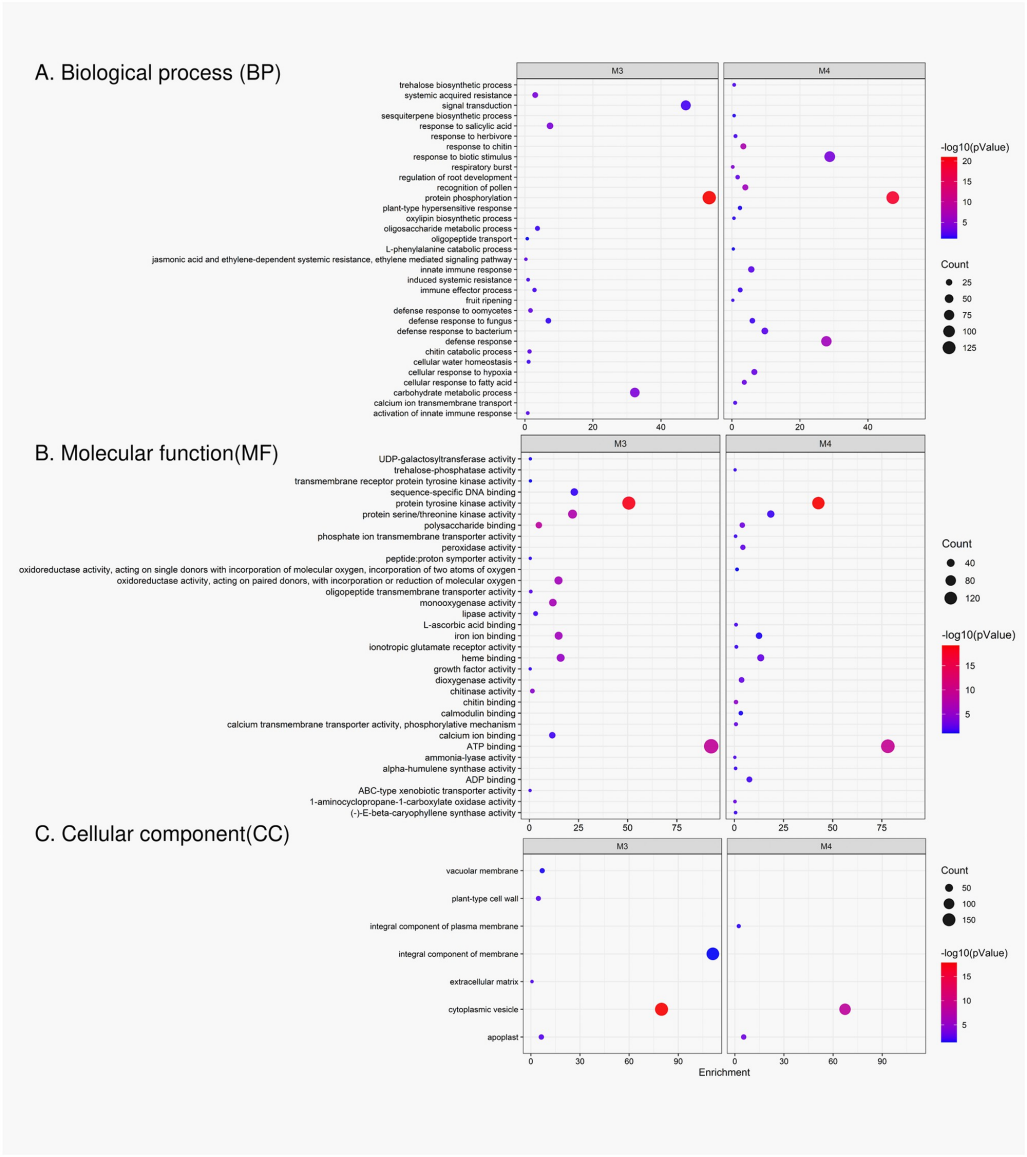
Gene ontology enrichment analysis of genes in modules 3 and 4. Significantly enriched gene ontology (GO) terms in M3 and M4 gene modules obtained from weighted gene co-expression network analysis (WGCNA) using differentially expressed genes (DEGs) in pecan cultivar Desirable when challenged with a pathogenic (De-Tif-11) or a non-pathogenic (Pa-OK-11) monoconidial *V*. *effusa* isolate. GO terms are categorized as (A) biological process DEGs involved (BP), (B) molecular function DEGs perform (MF) and (C) cellular component DEGs expressed (CC).

**Fig 8:**
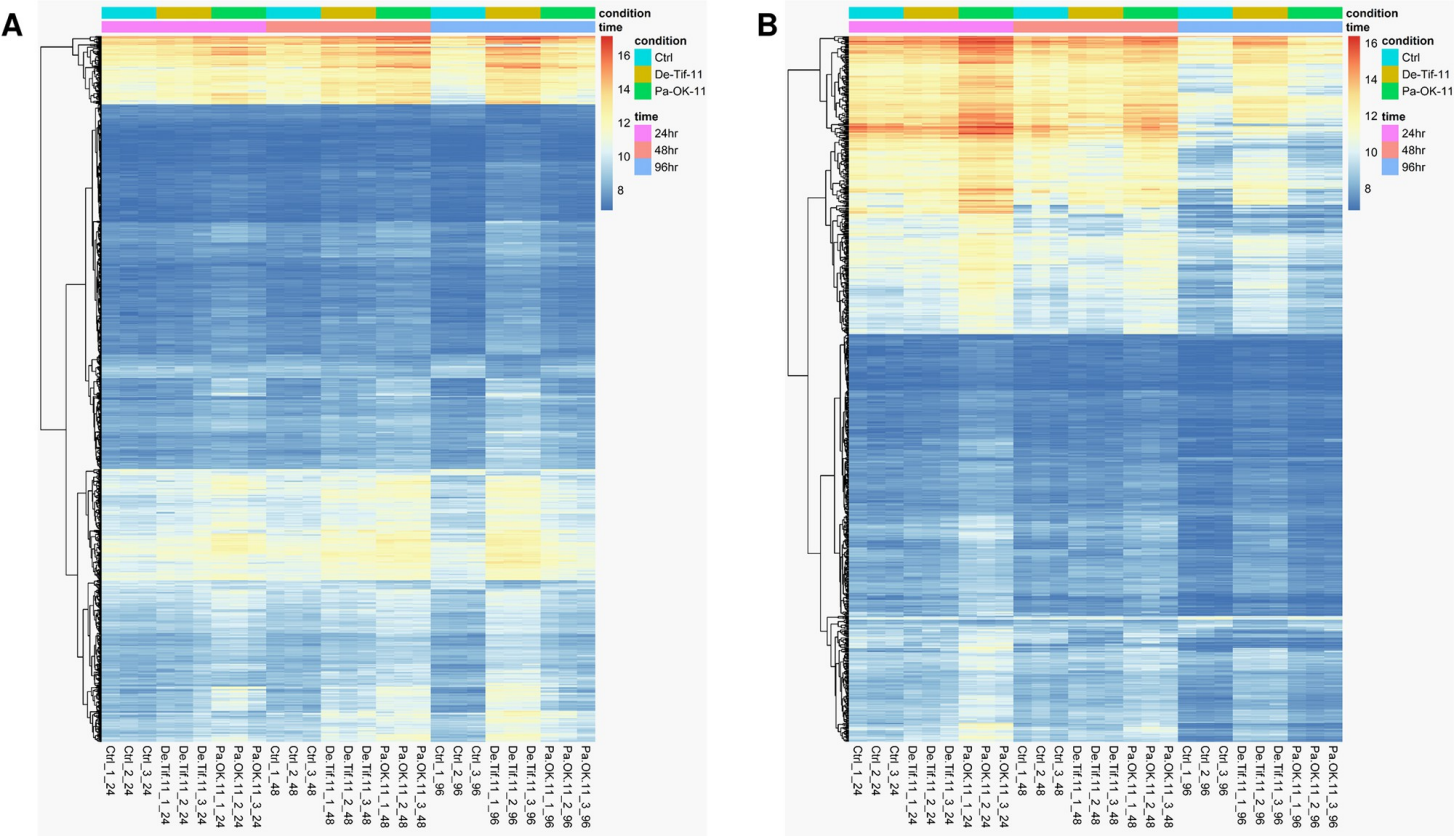
Heatmap showing expression pattern of genes in modules 3 and 4. Gene expression pattern in pecan cultivar ‘Desirable’ is shown across the control (Ctrl) and treatment samples of inoculation with a pathogenic (De-Tif-11) or a non-pathogenic (Pa-OK-11) monoconidial *V*. *effusa* isolate in three different time points (24, 48, and 96 hours post inoculation (hpi) within (A) module 3 and (B) module 4. Modules 3 and 4 are the results of weighted gene co-expression network analysis (WGCNA) using expression data of the genes that are differentially expressed in at least one sample of the study. Ctrl = control, De-Tif-11 = susceptible reaction, Pa-OK-11 = resistant reaction, hpi = hours post inoculation, 24hr = 2h hpi, 48hr = 48 hpi, 98hr = 96 hpi.

The 5,481 differentially expressed genes across the samples were clustered based on their correlated expression patterns, allowing 11 gene modules (denoted as M1 through M11) to be identified by weighted gene co-expression network analysis. The number of genes in each module ranged from 39 (M11) to 1,446 (M1) (Fig 6). Genes not significantly correlated to any gene modules were assigned to module zero, M0 (gray). Among 11 gene modules, gene ontology (GO) enrichment analysis showed that most of the genes related to disease resistance were clustered in M3 (brown) (S9 Table) and M4 (yellow) (S10 Table). GO enrichment analysis of M3 showed significantly enriched GO terms related to biological processes such as protein phosphorylation, defense response to fungus, signal transduction, immune effector process and carbohydrate metabolism (Fig 7). Genes in this module were associated with molecular functions such as protein tyrosine and serine/threonine kinase activities, ATP binding, oxidoreductase activity, polysaccharide binding, and chitinase activity. The M4 module had similarly enriched GO terms also found in M3, including protein phosphorylation mediated by protein tyrosine kinase activity, defense response to fungus, and immune effector process. However, M4 had more GO terms related to defense response than M3, including biological processes such as defense response, response to biotic stimulus, cellular response to hypoxia, respiratory burst, L-phenylalanine catabolic process, trehalose biosynthetic process, and response to chitin (Fig 7). Protein phosphorylation is an important cellular process mediated by protein kinase enzymes. These enzymes participate in activating several signaling cascades inside the cell to trigger resistance responses when under pathogen attack [35]. Significant enrichment of this biological process indicates the possible role of corresponding protein kinase coding genes in performing signaling activity under fungal attack and thereby triggering defense responses. Heatmaps of the expression pattern of genes in M3 and M4 showed that the expression levels of many genes belonging to these modules were higher in the resistant reaction (Pa-OK-11) at 24 and 48 hpi compared to the susceptible reaction (De-Tif-11) and the control (Fig 8). However, at 96 hpi, the susceptible reaction had relatively higher expression of these genes compared to resistant reaction and control. This gene expression pattern matches with the pattern of gene expression observed for differentially expressed genes related to the defense response at individual time points (Fig 5).

### Hub genes identification

**Fig 9:**
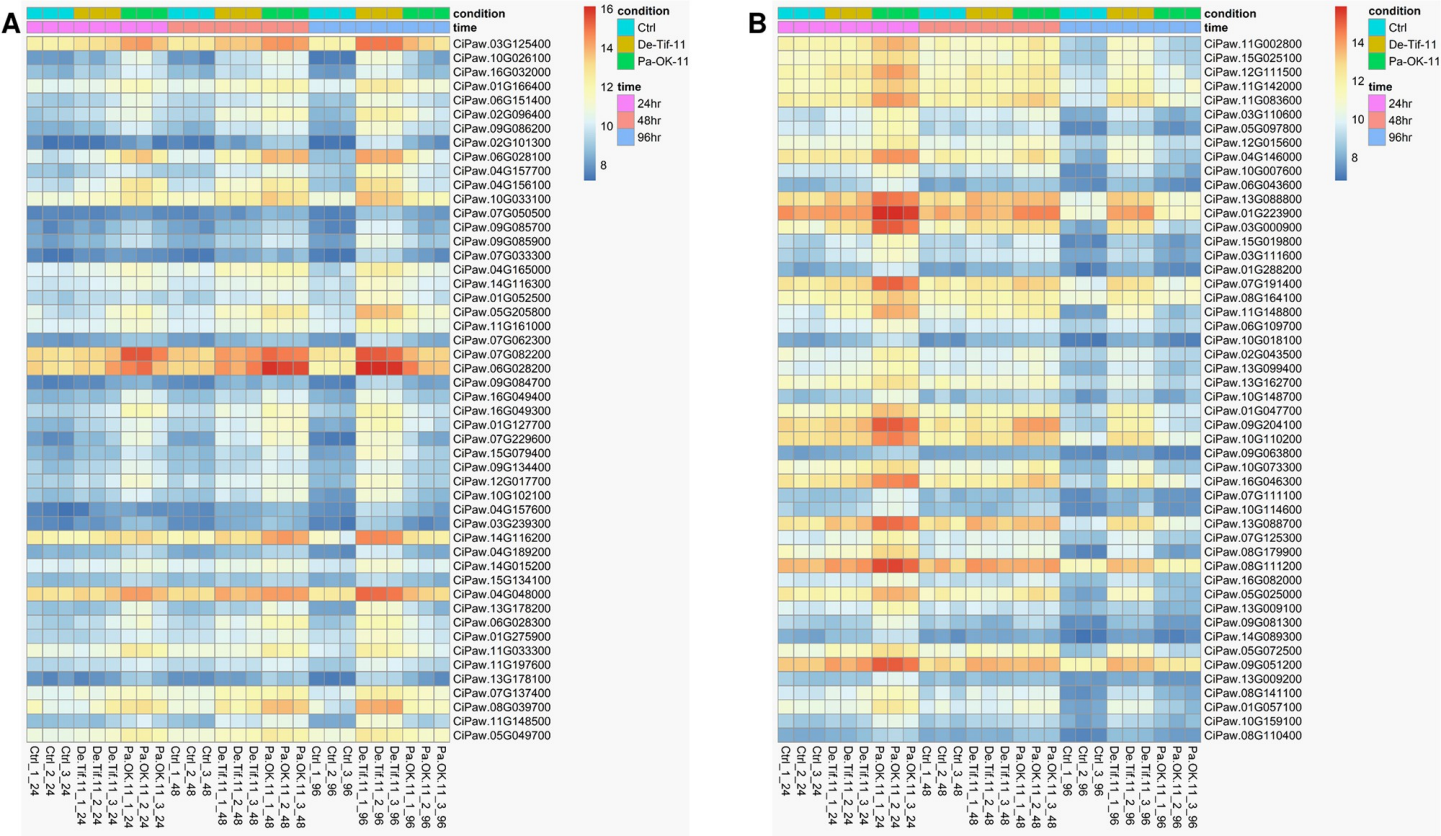
Heatmaps showing expression patterns of the top 50 hub genes in modules (A) 3 and (B) 4. The identification of gene modules 3 and 4 was achieved using weighted gene co-expression network analysis (WGCNA). Heatmaps were created using normalized read counts from RNA sequencing of leaflet samples obtained from the Desirable pecan cultivar. The samples encompass both control (Ctrl) and treatment groups at 24, 48, and 96 hours post inoculation (hpi) following inoculation with pathogenic (De-Tif-11) or non-pathogenic (Pa-OK-11) monoconidial *V*. *effusa* isolates. Ctrl = control, De-Tif-11 = susceptible reaction, Pa-OK-11 = resistant reaction, hpi = hours post inoculation, 24hr = 2h hpi, 48hr = 48 hpi, 98hr = 96 hpi.

Gene modules showing the enriched gene ontology terms related to defense response were further subjected to hub-gene analysis. Hub genes are those that have the highest level of interaction with other genes in the gene network. The top 50 hub genes were identified and ranked in modules 3 and 4 based on the number of edges connected to these genes in the corresponding co-expression gene networks (S1 Fig). The top 50 hub genes represent 5.07 and 6.32% of total genes present in module 3 and 4, respectively. The possible role of these hub genes in disease resistance was searched in a published gene annotation database [21]. Several hub genes in M3 were homologous to receptor-like protein kinases (Table 2) whereas hub genes in M4 represented diverse molecular functions related to disease resistance such as signal reception, signal transduction, biosynthesis and transport of plant secondary metabolites, and oxidoreductase activity. The gene expression pattern of these hub genes across the treatments and time points (Fig 9) is similar to the pattern observed in their corresponding gene modules (Fig 8). The expression level of hub genes was higher in the resistant reaction (Pa-OK-11) at 24 and 48 hpi compared to the susceptible reaction (De-Tif-11) and the control. At 96 hpi, the susceptible reaction had higher expression of these genes compared to the resistant reaction and the control treatments.

**Table 2:** Top 50 hub genes in modules 3 and 4. Hub genes were obtained from weighted gene co-expression network analysis. The ranking of hub genes was obtained from ‘degree’ method implemented in Cytoscape plugin, ‘cytoHubba’. Gene functional descriptions are derived from annotation database of ‘Pawnee’ primary reference genome.

Module	^a^Rank	Gene ID	Functional description
M3	1	CiPaw.03G125400	receptor-like protein kinase, putative, expressed
	2	CiPaw.10G026100	Concanavalin A-like lectin protein kinase family protein
	2	CiPaw.16G032000	Unknown
	4	CiPaw.01G166400	O-acetylserine (Thiol)-lyase/cysteine synthase or Cysteine Synthase
	5	CiPaw.06G151400	BRI1-associated receptor kinase
	6	CiPaw.02G096400	Copper amine oxidase family protein
	6	CiPaw.09G086200	(1 of 26) PTHR24362—SERINE/THREONINE-PROTEIN KINASE NEK
	8	CiPaw.02G101300	Unknown
	8	CiPaw.06G028100	Unknown
	8	CiPaw.04G157700	(1 of 6) PF08263—Leucine rich repeat N-terminal domain (LRRNT_2)
	11	CiPaw.04G156100	(1 of 6) PF08263—Leucine rich repeat N-terminal domain (LRRNT_2)
	11	CiPaw.10G033100	Insulinase (Peptidase family M16) family protein
	13	CiPaw.07G050500	Unknown
	13	CiPaw.09G085700	Protein tyrosine kinase
	13	CiPaw.09G085900	(1 of 26) PTHR24362—SERINE/THREONINE-PROTEIN KINASE NEK
	16	CiPaw.07G033300	Unknown
	17	CiPaw.04G165000	aldehyde dehydrogenase 2C4
	18	CiPaw.14G116300	Unknown
	19	CiPaw.01G052500	Unknown
	19	CiPaw.05G205800	2-oxoglutarate (2OG) and Fe(II)-dependent oxygenase superfamily protein
	21	CiPaw.11G161000	AAA ATPase
	22	CiPaw.07G062300	Eukaryotic aspartyl protease family protein
	23	CiPaw.07G082200	calreticulin 3
	23	CiPaw.06G028200	Unknown
	23	CiPaw.09G084700	Unknown
	26	CiPaw.16G049400	Unknown
	27	CiPaw.16G049300	Unknown
	27	CiPaw.01G127700	Unknown
	27	CiPaw.07G229600	Unknown
	27	CiPaw.15G079400	Unknown
	31	CiPaw.09G134400	(1 of 6) PF08263—Leucine rich repeat N-terminal domain (LRRNT_2)
	31	CiPaw.12G017700	Unknown
	31	CiPaw.10G102100	(1 of 6) PF08263—Leucine rich repeat N-terminal domain (LRRNT_2)
	31	CiPaw.04G157600	Unknown
	31	CiPaw.03G239300	Voltage-gated potassium channel
	36	CiPaw.14G116200	Unknown
	37	CiPaw.04G189200	(1 of 6) PF08263—Leucine rich repeat N-terminal domain (LRRNT_2)
	37	CiPaw.14G015200	Malate dehydrogenase, decarboxylating
	37	CiPaw.15G134100	Unknown
	37	CiPaw.04G048000	Glutamine synthetase
	41	CiPaw.13G178200	Unknown
	41	CiPaw.06G028300	Unknown
	41	CiPaw.01G275900	IQ-domain 3
	44	CiPaw.11G033300	alpha/beta-Hydrolases superfamily protein
	44	CiPaw.11G197600	PLC-like phosphodiesterase family protein
	44	CiPaw.13G178100	Unknown
	44	CiPaw.07G137400	(1 of 26) PTHR24362—SERINE/THREONINE-PROTEIN KINASE NEK
	44	CiPaw.08G039700	plant natriuretic peptide A
	44	CiPaw.11G148500	Cytochrome P450, CYP4 superfamily
	50	CiPaw.05G049700	Unknowns
M4	1	CiPaw.11G002800	Unknown
	2	CiPaw.15G025100	wall-associated kinase 2
	3	CiPaw.12G111500	Leucine-rich repeat transmembrane protein kinase
	4	CiPaw.11G142000	hydroxymethylglutaryl-CoA synthase
	5	CiPaw.11G083600	LRk-type protein, putative, expressed
	6	CiPaw.03G110600	(1 of 5) PTHR22849//PTHR22849:SF39—WDSAM1 PROTEIN // SUBFAMILY NOT NAMED
	7	CiPaw.05G097800	(1 of 1) PTHR33052:SF10—F3H9.15 PROTEIN
	8	CiPaw.12G015600	ABC transporter, ATP-binding protein, putative, expressed
	9	CiPaw.04G146000	Unknown
	10	CiPaw.10G007600	GDSL-like lipase/acylhydrolase, putative, expressed
	11	CiPaw.06G043600	AAA-type ATPase family protein, putative, expressed
	12	CiPaw.13G088800	Tropine dehydrogenase/reductase
	13	CiPaw.01G223900	Fructan fructosyltransferase
	14	CiPaw.03G000900	Unknown
	15	CiPaw.15G019800	(1 of 6) PF08263—Leucine rich repeat N-terminal domain (LRRNT_2)
	16	CiPaw.03G111600	Ankyrin repeat family protein
	17	CiPaw.01G288200	Calcium-dependent lipid-binding (CaLB domain) family protein
	18	CiPaw.07G191400	4-coumarate: CoA ligase 1
	19	CiPaw.08G164100	lipoxygenase, putative, expressed
	20	CiPaw.11G148800	Cytochrome P450, CYP4 superfamily
	21	CiPaw.06G109700	Sec14p-like phosphatidylinositol transfer family protein
	22	CiPaw.10G018100	plant U-box 24
	23	CiPaw.02G043500	cysteine-rich RLK (RECEPTOR-like protein kinase) 29
	24	CiPaw.13G099400	domain of unknown function DUF966 domain containing protein, expressed
	25	CiPaw.13G162700	ADP/ATP carrier 1
	26	CiPaw.10G148700	Protein of unknown function (DUF3411)
	27	CiPaw.01G047700	alpha/beta-Hydrolases superfamily protein
	28	CiPaw.09G204100	PAR1 protein
	29	CiPaw.10G110200	reticuline oxidase-like protein precursor, putative, expressed
	30	CiPaw.09G063800	Protein kinase superfamily protein
	31	CiPaw.10G073300	WRKY DNA-binding protein 31
	32	CiPaw.16G046300	phospholipase A 2A
	33	CiPaw.07G111100	Unknown
	34	CiPaw.10G114600	S-locus lectin protein kinase family protein
	35	CiPaw.13G088700	Tropine dehydrogenase/reductase
	36	CiPaw.07G125300	MDR-like ABC transporter, putative, expressed
	37	CiPaw.08G179900	Unknown
	38	CiPaw.08G111200	Zinc-binding dehydrogenase family protein
	39	CiPaw.16G082000	NB-ARC domain containing protein, expressed
	40	CiPaw.05G025000	(1 of 6) PF08263—Leucine rich repeat N-terminal domain (LRRNT_2)
	41	CiPaw.13G009100	Unknown
	42	CiPaw.09G081300	expressed protein
	43	CiPaw.14G089300	(1 of 26) PTHR24362—SERINE/THREONINE-PROTEIN KINASE NEK
	44	CiPaw.05G072500	Unknown
	45	CiPaw.09G051200	cytochrome P450, family 98, subfamily A, polypeptide 3
	46	CiPaw.13G009200	Unknown
	47	CiPaw.08G141100	UDP-Glycosyltransferase superfamily protein
	48	CiPaw.01G057100	Unknown
	49	CiPaw.10G159100	ammonium transporter 2
	50	CiPaw.08G110400	Unknown

^a^Hub genes with equal number of connection (‘degree’) in the gene network are given same rank.

### Measuring candidate gene expression with RT-qPCR

**Fig 10:**
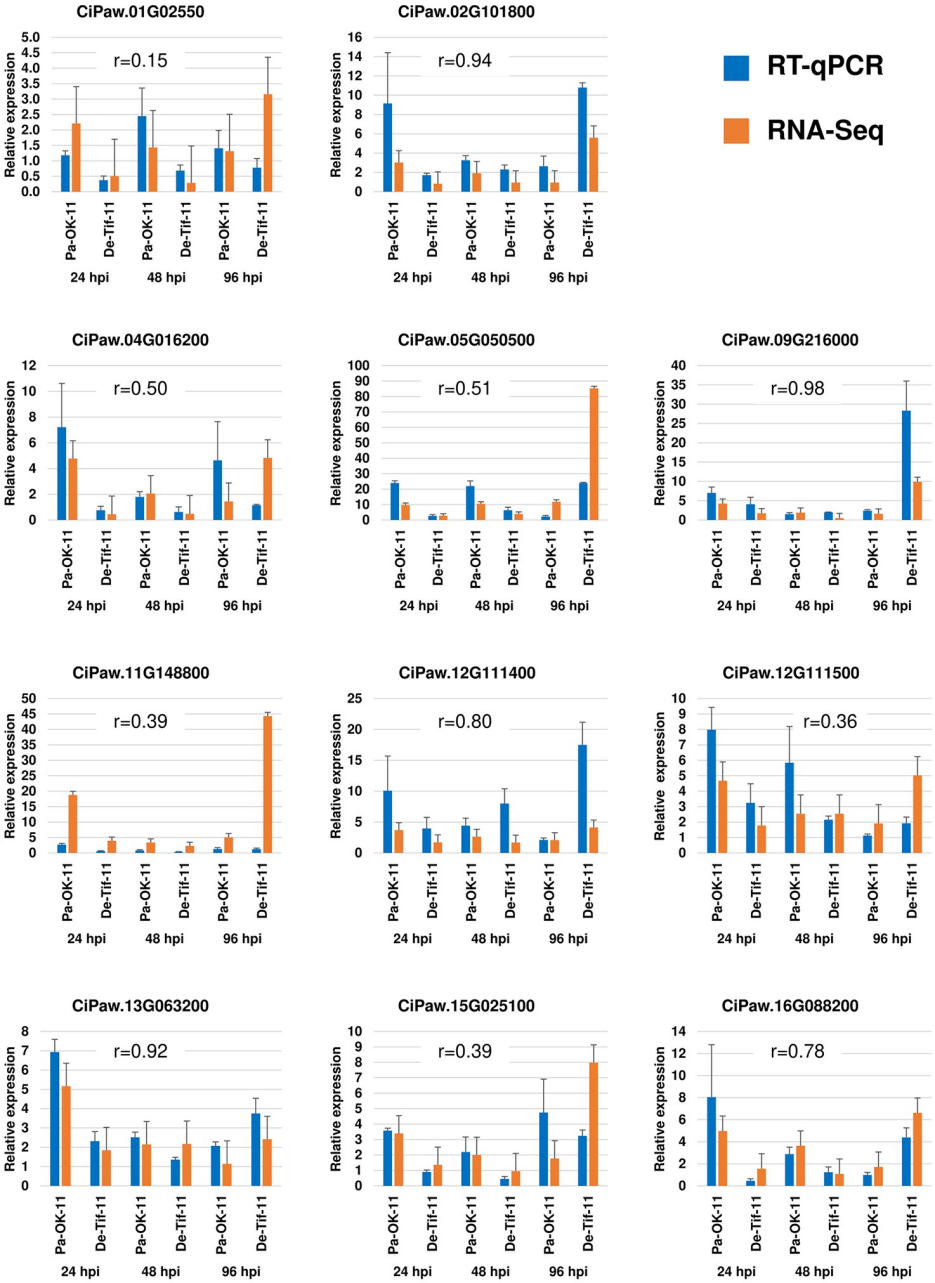
RT-qPCR of defense-related genes. Bar graph showing mean ± standard error of fold change in expression of defense-related genes under resistant (Pa-OK-11) and susceptible (De-Tif-11) reactions at three different time points (24, 48, and 96 hours post inoculation (hpi)) after inoculation of pecan leaflets with a pathogenic or a non-pathogenic isolate of *Venturia effusa*. The blue and orange bars represent relative gene expression obtained by RT-qPCR and RNA-Seq-based differential gene expression analysis, respectively. The correlation coefficient (r) represents the correlation of gene expression between the RT-qPCR and RNA-Seq studies. Eleven genes used in RT-qPCR study for validation of RNA-Seq results were CiPaw.01G025500 (Disease resistance protein (CC-NBS-LRR class) family)), CiPaw.02G101800 (CHIT2—Chitinase family protein precursor), CiPaw.04G016200 (Calcium-binding EF-hand family protein), CiPaw.05G050500 (CHIT7—Chitinase family protein precursor), CiPaw.09G216000 (WRKY family transcription factor), CiPaw.11G148800 (flavonoid 3’-monooxygenase CYP75B137-like), CiPaw.12G111400 (Leucine-rich repeat transmembrane protein kinase), CiPaw.12G111500 (probable LRR receptor-like serine/threonine-protein kinase), CiPaw.13G063200 (trans-cinnamate 4-monooxygenase-like), CiPaw.15G025100 (putative wall-associated receptor kinase-like 16), and CiPaw.16G088200 (Calmodulin binding protein-like).

Eleven genes related to the defense response against fungi were randomly selected to perform real-time quantitative PCR (RT-qPCR). These eleven genes were CiPaw.01G025500 (Disease resistance protein (CC-NBS-LRR class) family)), CiPaw.02G101800 (CHIT2—Chitinase family protein precursor), CiPaw.04G016200 (Calcium-binding EF-hand family protein), CiPaw.05G050500 (CHIT7—Chitinase family protein precursor), CiPaw.09G216000 (WRKY family transcription factor), CiPaw.11G148800 (flavonoid 3’-monooxygenase CYP75B137-like), CiPaw.12G111400 (Leucine-rich repeat transmembrane protein kinase), CiPaw.12G111500 (probable LRR receptor-like serine/threonine-protein kinase), CiPaw.13G063200 (trans-cinnamate 4-monooxygenase-like), CiPaw.15G025100 (putative wall-associated receptor kinase-like 16), and CiPaw.16G088200 (Calmodulin binding protein-like). Relative expression of these genes obtained from real-time quantitative PCR was highly correlated with their log2fold change estimated from differential gene expression analysis using RNA-Seq data (Fig 10). The correlation coefficients (r) ranged from 0.15 to 0.98. The RT-qPCR results of most of the tested genes followed a very similar expression pattern observed in RNA-Seq (Fig 10, S2 Fig).

## Discussion

The quality and reliability of RNA-Seq results are fundamentally dependent on the quality of sequence data generated. After removing reads that mapped to the SILVA rRNA database, the percentage of the remaining pair-end reads uniquely mapped to the ‘Pawnee’ reference genome ranged from 85.2 to 94.3% (average 91.4%). This result is higher or very close to the percentage of read mapped reported in other transcriptomic studies in fruit crops such as 78.8–80.9% in pear [36], 78.4–81.3% in apple [37], 91% in grape [38] and 92–93% in peach [39]. A high mapping percentage of RNA-Seq reads generally indicates a high quality of reference genome and read sequences as well as a less diverged reference genome from the genotype being studied. One of the parents of ‘Desirable’, ‘Success’, is a grandparent of ‘Pawnee’ [16] indicating close genetic relationship between these two cultivars. We also performed real-time quantitative PCR (RT-qPCR) on a subset of defense-related genes. The high correlation between RT-qPCR and RNA-Seq data confirms the reliability of the results from transcriptomic analysis. Similar correlation coefficients have been reported in other studies, such as 0.76–0.94 in apple [40], 0.623 (mean) in table grape [41], and 0.36–0.96 in pecan [42].

The substantial differences were observed between the amount of infection as measured by the percentage of subcuticular hyphae in ‘Desirable’ leaf tissue caused by two different fungal isolates highlights that the host responded quite differently to the contrasting isolates. This further supports the previous observations of host specificity of the scab pathogen [6, 18] and clearly shows the importance of studying and understanding the pathotype-specific host resistance system of pecans. *V*. *effusa* conidia germinate, form germ tubes and appressoria, and penetrate the pecan leaf cuticle within 12 hpi [43]. Cuticle penetration occurs on both susceptible and resistant pecan leaves, but subcuticular hyphae grow copiously only in the susceptible host [18, 44]. In contrast, the resistant reaction is highlighted by the arrest of further subcuticular growth and the presence of dark staining underneath the appressoria by 96 hpi [18]. Thus, resistance to *V*. *effusa* appears to be controlled by host mechanisms occurring after cuticle penetration and prior to 96 hpi. The number of differentially expressed genes was markedly higher in the resistant reaction compared to the susceptible reaction at 24 hpi. This intense transcriptomic response corroborates that the resistance reaction against the invading fungus in pecan starts early and is likely triggered by germinating conidia penetrating into the sub-cuticular space. A continuous decline in the number of differentially expressed genes was observed at 48 and 96 hpi in resistant reaction. This suggests that as soon as pathogen is warded off in the subcuticular space close to the cell wall, the fugal hyphae stop growing and no longer trigger the host resistance response. This explains the more similar transcriptomic state of the resistant and control treatments at 96 hpi. In contrast, the delayed transcriptomic response observed in the susceptible reaction (96 hpi) is likely insufficient to contain fungal growth. This differential gene expression pattern in the resistant and susceptible reactions indicates that expression of defense-related genes at the early stage of pathogen invasion/growth may be vital in producing resistance to the scab pathogen. Hence, conducting a time series study in this case was justified and vital to understand how and when the host responds to fungal isolates which differ in their capacity to cause infection.

Gene ontology (GO) enrichment analysis further supported the role of early defense responses. Biological processes related to defense response, response to biotic stimulus, immune effector processes, and defense response to oomycetes were significantly enriched and attributed to upregulated genes at 24 and 48 hpi in the resistant reaction. These processes were absent or only appeared later in the susceptible reaction, underscoring their possible importance in conferring scab resistance. GO terms related to plant type cell-wall biogenesis, cell wall modification, cell wall thickening, and primary cell wall biogenesis were significantly enriched at 96 hpi in the susceptible reaction, and genes that belong to these enriched GO terms were downregulated. Downregulation of these genes in susceptible reaction may produce a compromised state of cell wall development, allowing fungal penetration of the intercellular matrix. Additionally, the L-phenyl alanine catabolic and lignin biosynthetic processes were significantly enriched at 24 hpi in the resistant reaction, and genes involved in these pathways were upregulated. This may indicate that an early resistance response by the host results in the increased lignification of the cell wall, creating a protective barrier against the invading fungus. Subcuticular hyphae are formed only in immature leaves of susceptible pecan genotypes [44], whereas mature leaves of pecan are ontologically resistant to the scab infection. Premature lignification near penetration sites in response to scab infection in immature leaves is a possible mechanism contributing to resistance.

The molecular mechanisms of host resistance against biotic stressors are complex. In biological systems, the simultaneous activation or regulation of genes involved in various cellular processes, such as signal reception, signal transduction, and transcriptional activation of defense-related genes, can happen in a cascade to trigger the resistance response [45–47]. Weighted gene co-expression network analysis studies are particularly helpful in time series experiments in which the pattern of gene expression provides an overall view of how the molecular mechanisms operate in making the host susceptible or resistant to the invading pathogen. We employed WGCNA and identified 11 different gene modules, two of which were associated with disease resistance. Modules M3 and M4 had genes related to defense response, protein phosphorylation, signal transduction, cell wall modification, and biosynthesis of plant secondary metabolites. The enrichment of these modules with defense-related genes suggests their role in mediating the host’s response to scab infection. Gene expression patterns in M4 was very similar to that observed in GO enrichment analysis of up-and downregulated genes at three different time points separately.

One of the important basal defense mechanisms in plants is response triggered by pathogen associated molecular patterns (PAMPs). This leads to PAMP-triggered immunity. Genes encoding nucleotide binding (NBS) leucine-rich repeats (LRR) domain-containing proteins are the most studied class for their role in PAMP-triggered immunity in plants. NBS-LRR are categorized into TIR-NBS-LRR class, those containing an amino-terminal domain with homology to the Toll and interleukin 1 receptors, and non-TIR class. The most studied genes among non-TIR class are genes containing α-helical coiled-coil–like (CC) sequences in their amino-terminal domain (CC-NBS-LRR) [48, 49]. CC-NBS-LRR is found in both monocots and dicots while TIR-NBS-LRR are only present in dicots [50–53]. The diverse recognition capabilities of NBS-LRRs are closely associated with the highly variable LRR region, allowing them to identify a wide array of pathogen-derived products. Both TIR-NBS-LRR and CC-NBS-LRR coding genes have been characterized in several crops including apple [54–57], cotton [58, 59], grapes [60, 61], and tomato [62]. Receptor-like kinases (RLKs) are an important class of cell surface proteins that play a crucial role in signal transduction through phosphorylation reactions. Structurally, RLKs are typically composed of an extracellular ligand-binding domain, a transmembrane domain, and an intracellular kinase domain. The extracellular RLK domain can bind with variety of ligands such as PAMP, degraded pathogen or cell wall products, or effector molecules [35, 63, 64]. The intracellular kinase domain is responsible for phosphorylating downstream signaling proteins. Several RLK class genes have been reported to play an important role in disease resistance in a wide range of crops including tomato [65], tobacco [66], rice [67–69], apple [70, 71], citrus [72], maize [73], and wheat [74–76]. Many genes found in both M3 and M4 display homology with RLKs and NBS-LRRs encoding genes (S9 and S10 Tables), suggesting their potential involvement in signaling and the activation of PAMP-triggered immunity during pecan scab pathogen attack.

Several classes of transcription factors (TF) involved in defense mechanisms in plants have been reported. Both gene modules (M3 and M4) had several genes related to transcription factors, particularly those belonging to WRKY and MYB families. WRKY transcription factors are one of the largest families of transcriptional regulators commonly found in plants and their role have been associated with transcriptional regulation of defense related gene expression [77]. WRKY TF has been linked to both positive and negative regulation of defense response in barley [78, 79], wheat [80], rice [81], cotton [82], tomato [83, 84], and apple [85, 86]. The MYB TF family is another important TF in plants and reported for their involvement in cuticular wax biosynthesis [87] and the salicylic acid pathway [88] in apple, enhanced H_2_O_2_ accumulation, pathogenesis-related (PR) gene expression, and salicylic acid signaling pathway-induced cell death in wheat [80], and defense-induced lignification in pear [89]. The differential expression of a number of WRKY and MYB domain transcription factor related genes in WGCNA modules M3 and M4 suggests their potential involvement in regulating gene expression for scab resistance in pecan.

The plant’s primary cell wall is principally made up of carbohydrate-based materials including cellulose, pectin, and hemicellulose. The secondary cell wall also has cellulose but has more lignin and xylan content [90]. Biogenesis and modification of cell wall components have been described as important defense mechanisms against fungal attack as the cell wall serves as the first physical and defensive barrier against the pathogen [91]. Lignin biosynthesis mediated by genes encoding phenylalanine ammonia lyase, cinnamic alcohol dehydrogenase, and peroxidase enzymes is found to be involved in disease resistance in pummelo fruit [92]. Induced expression of lignin biosynthesis genes has also been reported in wheat when challenged by the fungus *Fusarium graminearum* [93]. Intergenic transfer of the major apple scab (*V*. *inequalis*) resistance gene, *Rvi6*, into pear resulted in increased lignification which indicates role of lignification in scab resistance [15]. Several other studies have shown a correlation between cellular lignification and disease resistance in tobacco [94], soybean [95], jujube fruit [96], and Reed canary grass [97]. Several genes related to lignin and cellulose biosynthesis were present in M3 and M4. Examples were genes encoding 4-coumarate—CoA ligase, a key enzyme in secondary cell wall development and lignin synthesis [98], laccase precursor protein [99], and phenylalanine ammonia-lyase [100].

Cytochrome P450 (CYP450) enzymes play a crucial role in disease resistance in plants by regulating the synthesis of different secondary compounds. These compounds include phytoalexins, terpenoids, and flavonoids. A CYP716A16 gene that belongs to the CYP450 protein family was identified to be involved in resistance against necrotrophic *Rhizoctonia solani* and hemibiotrophic *Xanthomonas oryzae* pv. *oryzae* in rice [101]. Transcriptomic and metabolomic studies showed that the overexpression of CYP716A16 activated flavonoid biosynthesis and increased the amounts of narcissoside, methylophiopogonanone A, oroxin A, and amentoflavone in plants. The number of cytochrome P450 related genes were markedly high in module 3 and 4. A previous metabolomic study of two pecan cultivars ‘Kanza’ and ‘Pawnee’ indicated the potential involvement of quercetin derivatives in resistance against scab [102]. A transgenic study of apple *Rvi6* also indicated the involvement of flavonoids, more specifically quercetin and kaempferol, in pear scab resistance.

Several genes related to chitinase and chitin elicitor receptor kinase were detected in M3 and M4. Chitinase hydrolyzes chitin, the second most abundant polymer found in living organisms after cellulose, and the major component of fungal cell walls. Studies have shown the potential role of chitinases in defense against fungal pathogens. In apple, increased expression of a WRKY transcription factor was positively correlated to endochitinase activity [95] and results revealed the interaction between WRKY transcription factor and the promoter region of the endochitinase gene. Lab-based chitin treatment of pecan leaves of the cultivars ‘Excel’ and ‘Pawnee’ have shown early induction of several chitinase coding genes and genes involved in the signaling cascade indicating that they may have important roles in defense against fungal pathogens at early infection stages [12].

The top 50 hub genes identified in gene modules M3 and M4 were related to several signaling and defense response genes, particularly receptor-like protein kinase, ABC transporter, 4-coumarate: CoA ligase, Cytochrome P450, WRKY DNA-binding protein etc. The expression pattern of these hub genes aligns with the overall expression pattern observed in the corresponding gene modules.

## Conclusion

This comparative transcriptomic analysis provided an in-depth insight into the molecular mechanisms underlying scab resistance in pecans. A clear difference in transcriptomic response by the host was detected when inoculated with scab isolates that vary in their pathogenicity to infect and cause disease. The findings show the importance of early defense responses in pecan triggered by germinating conidia in the sub-cuticular space. The networks of genes related to scab resistance were identified using weighted gene co-expression network analysis. The identification of key gene modules and hub genes associated with disease resistance opens avenues for further research into the specific genes and pathways involved in pecan’s defense against scab. Future work can use other omics approach such as metabolomics to generate metabolite profiles and establish correlations with the gene expression profiles obtained in this study to narrow down downstream biological pathways and genes involved in scab resistance in pecan.

## Supporting information

S1 File Scripts used to perform bioinformatic analysis. (TXT)

S1 Fig Top 50 hub genes in modules 3 (M3) and 4 (M4). Each circle in the gene network represents one of top 50 hub genes (nodes) and color intensity for the gene nodes is arranged in decreasing order of degree, the darkest color represents the highest degree.
(PNG)

S2 Fig Correlation of gene expression obtained from RT-qPCR and RNA-Seq studies. (TIFF)

S1 Table Forward and reverse primer sequence used for the RT-qPCR. (XLSX)

S2 Table Treatment conditions, biological replicates, and mapping statistics of RNA-sequencing reads. (XLSX)

S3 Table Differentially expressed genes in resistant reaction (Pa-OK-11) at 24 hpi. (XLSX)

S4 Table Differentially expressed genes in resistant reaction (Pa-OK-11) at 48 hpi. (XLSX)

S5 Table Differentially expressed genes in resistant reaction (Pa-OK-11) at 96 hpi. (XLSX)

S6 Table Differentially expressed genes in susceptible reaction (De-Tif-11) at 24 hpi. (XLSX)

S7 Table Differentially expressed genes in susceptible reaction (De-Tif-11) at 48 hpi. (XLSX)

S8 Table Differentially expressed genes in susceptible reaction (De-Tif-11) at 96 hpi. (XLSX)

S9 Table Gene ID, related function, and log2fold change of genes in module 3 (M3). (XLSX)

S10 Table Gene ID, related function, and log2fold change of genes in module 4 (M4). (XLSX)

S11 Table Gene ontology enrichment analysis of up and downregulated genes at 24, 46, and 96 hpi. (XLSX)

S12 Table Raw read counts across three biological replications for control, resistant (Pa-OK-11), and susceptible (De-Tif-11) reactions. (XLSX)
