# Supplementary figures and images for: Transcriptome analysis under pecan scab infection reveals the molecular mechanisms of the defense response in pecans
Source: PLoS One. 2024 Nov 21;19(11):e0313878. doi: 10.1371/journal.pone.0313878 (PMC11581225; doi:10.1371/journal.pone.0313878)

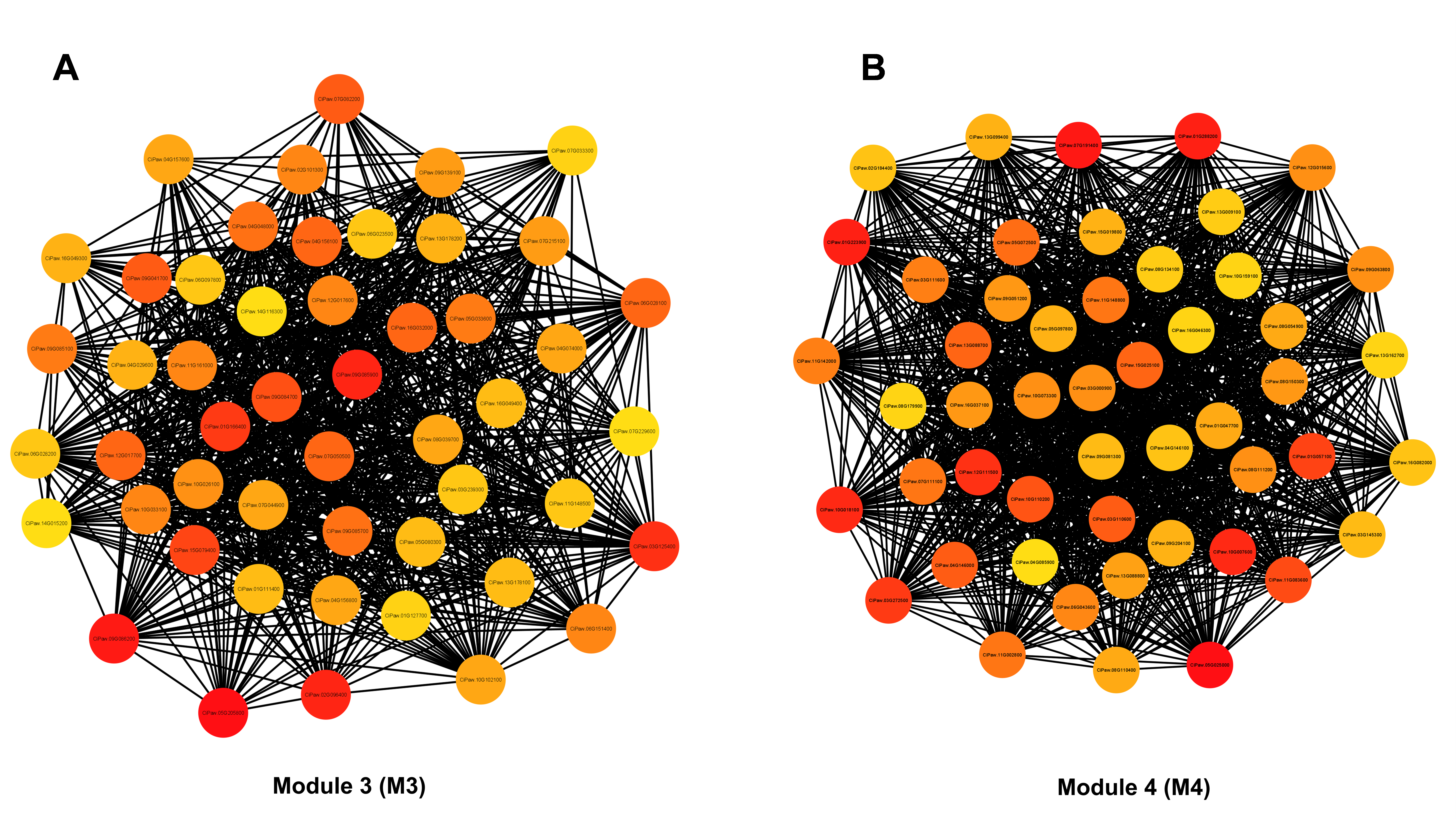

Supplement: S1 Fig — Each circle in the gene network represents one of top 50 hub genes (nodes) and color intensity for the gene nodes is arranged in decreasing order of degree, the darkest color represents the highest degree. (PNG) [file pone.0313878.s002.png]

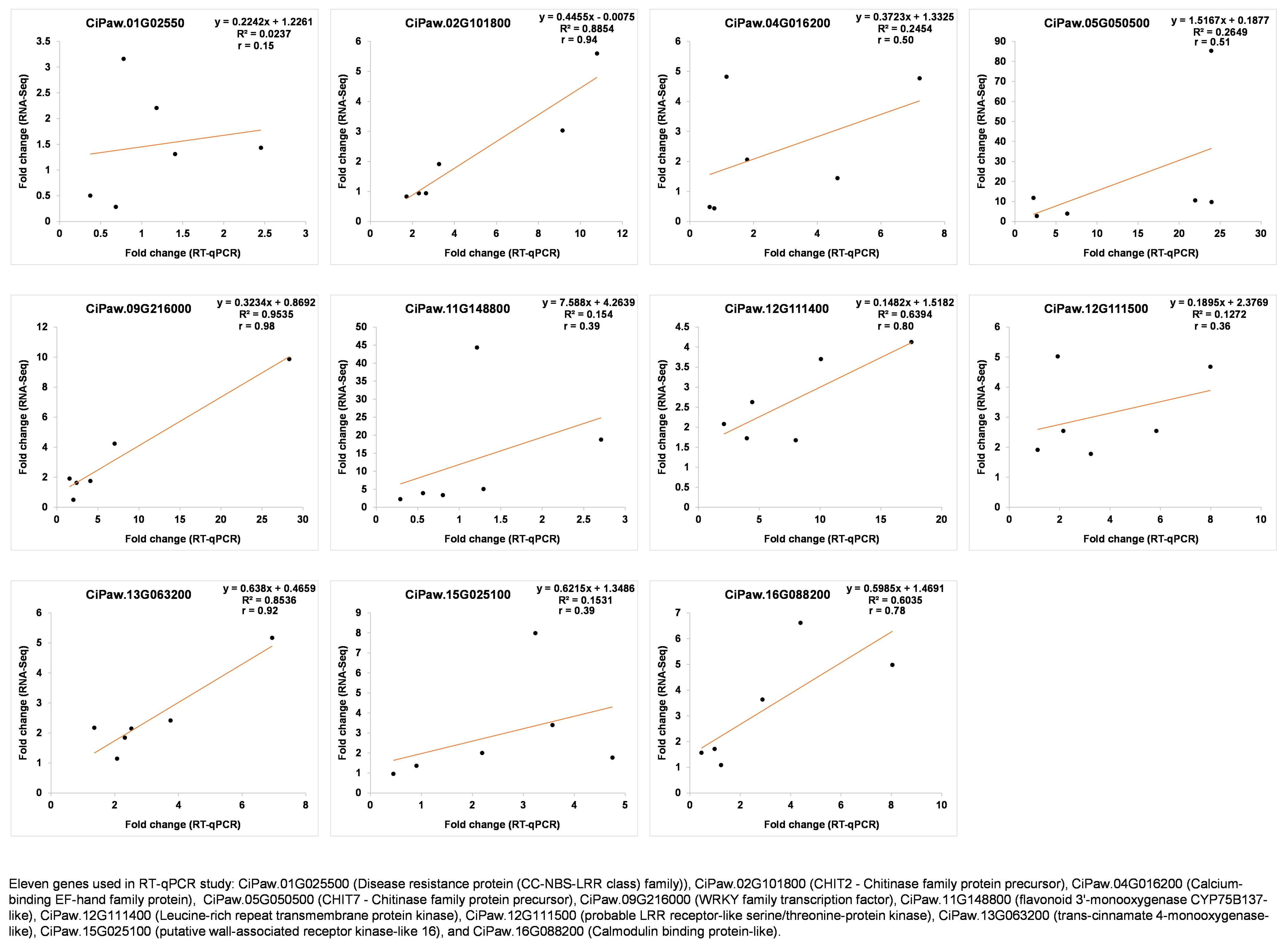

Supplement: S2 Fig — (TIFF) [file pone.0313878.s003.tiff]
